# Effect of *Erica* sp. Honey against Microorganisms of Clinical Importance: Study of the Factors Underlying this Biological Activity

**DOI:** 10.3390/molecules18044233

**Published:** 2013-04-11

**Authors:** Xesus Feás, Antonio Iglesias, Sandra Rodrigues, Leticia M. Estevinho

**Affiliations:** 1 Department of Organic Chemistry, Faculty of Science, University of Santiago de Compostela, E-27002 Lugo, Galicia, Spain; E-Mail: xesus.feas@usc.es; 2 Department of Anatomy and Animal Production, Faculty of Veterinary, University of Santiago de Compostela, E-27002 Lugo, Galicia, Spain; E-Mail: antonio.iglesias@usc.es; 3 CIMO-Mountain Research Center, Agricultural College of Bragança, Polytechnic Institute of Bragança, Campus Santa Apolónia, E 5301-855, Bragança, Portugal; E-Mail: srodrigues@ipb.pt

**Keywords:** antibacterial properties, antifungal properties, heather honey, hydrogen peroxide, polyphenols, sugars

## Abstract

This study aimed to determine the factors (phenolic compounds, flavonoids, sugars or H_2_O_2_) that contribute the most to the antimicrobial activity of heather honey samples against four yeasts and four bacteria with medical importance. To discard the effect of H_2_O_2_ in the antimicrobial activity, catalase was added. To evaluate the osmotic pressure’s effect, artificial honey was also used. Phenolic compounds and flavonoids were determined and Pearson’s correlation analysis was performed to assess whether these correlated with antimicrobial activity. The amount of phenolic compounds ranged from 630.89 ± 5.21 GAE kg^−1^ to 718.92 ± 4.41 GAE kg^−1^, while the flavonoids varied between 450.72 ± 5.67 CAE kg^−1^ and 673.98 ± 4.33 CAE kg^−1^. For the bacteria, the minimum inhibitory concentration (MIC) of the honey without catalase ranged from 1.01 ± 0.50% to 10.00 ± 4.72% and was between 2.00 ± 0.94% and 13.27 ± 5.23% for honey with catalase. Concerning the yeasts, the MICs was between 13.16 ± 4.08% and 20.00 ± 5.09% for honey without catalase and between 14.95 ± 4.16% and 25.67 ± 5.50% for honey with catalase. The elucidation of the antimicrobial factors and action mechanisms is essential for the correct use of honey in therapeutic applications.

## 1. Introduction

The treatment of bacterial infections is increasingly complicated by the ability of bacteria to develop resistance to current antimicrobial agents [1]. In addition, the emergence of resistant pathogenic yeasts, the adverse effects and the limited spectrum of action of the currently available drugs, justifies the need for less and better use of antibacterials and antifungals, improved infection control, and research on new therapeutic compounds [2,3].

Along with the rapidly increasing research into natural health remedies, there has been a resurgence of interest in the therapeutic use of honey. Although knowledge of honey use was widespread in ancient civilizations not only as a source of carbohydrates and sweetener, but also for medicinal purposes, today modern science has made it possible to specify their medical significance as bactericidal and bacteriostatic [4,5], nematicidal [6], antiviral [7], antifungal [8,9], antioxidant [10,11] and antitumoral [12]; thus, this bee product has been demonstrated to be a suitable alternative for healing wounds, burns and various skin conditions [13,14].

Bees make honey, but not all honey is equal. In fact, even when they have common compositions and physicochemical properties (high osmolarity, low moisture, and acidity), the antimicrobial potency of different honeys can vary by more than 100-fold [15]. There are some factors that are closely related to the antibacterial capacity of honey, among which the level of hydrogen peroxide determined by relative levels of glucose oxidase and catalase contributes most [16]. Others are based on its geographical, seasonal and botanical origin as well as the harvesting, processing and storage conditions [17,18].

It was stated that heather (*Erica* sp.) honeys exhibit non-Newtonian, shear-thinning behavior with a tendency to yield stress and that they are thixotropic [19]. Heather honey is a valuable cure: it is used in therapy for cases of inflammation of the throat or the mucous membrane of the oral cavity. It is recommended for the therapy of urinary tract infections, prostate diseases, nephrolithiasis and enteritis, as well [19]. In fact, *Ericaceae* family is often used in folk medicine as an alternative therapeutic tool to treat hyperlipidaemia, as a diuretic, astringent, antiseptic and in the treatment of urinary infections [20,21,22].

The literature reviewed shows us that research on monofloral *Erica* sp. honeys is mainly focused on: their physicochemical and palynological features [23,24]; the assessment of the possible markers for their floral origin [25,26]; and, more recently, the antioxidant and antibacterial properties of the phenolic compounds [27,28], and the antifungal potential found in Portuguese heather honeys [29] which have demonstrated promising results.

The healing properties of honey depend largely on the floral source with which honeybees are nourished. Recent research in nectar chemistry shows us that nectar is more than water and sugars. The biochemical functions and different substances of nectar act as protection from microbial infestation through a novel biochemical pathway called the “Nectar Redox Cycle” [30]. *Erica* species contain many active substances such as flavonoids, anthocyanidols, coumarins and triterpenic compounds, which are expected to be found in heather honey. Moreover, the major compounds of *Erica* sp flowers have been isolated and proven to have antimicrobial [31] and antiulcer activities [32] as well as cytotoxic and anticarcinogenic properties [33].

The aim of the present paper was to assess the *in vitro* antimicrobial and antifungal properties of heather honey against clinically important bacterial and fungal species, including *Bacillus* *cereus*, *Pseudomonas aeruginosa*, *Staphylococcus* *aureus*, *Escherichia* *coli*, *Candida famata*, *Candida albicans*, *Candida krusei* and *Cryptococcus neoformans*, in the absence or presence of catalase. Furthermore, correlations among phenolics and flavonoids and antimicrobial properties of honeys were studied. As far as we know, this is the first comprehensive study on the factors underlying the antibacterial and antifungal activities of heather honey.

## 2. Results and Discussion

### 2.1. Pollen Analysis

The amount of pollen grains from *Erica sp*. per sample varied between 59.3 ± 1.2% and 72.0 ± 2.0%, with the mean value of 69.5 ± 4.0%. Pollen grains of *Lavandula* spp., *Prunus* spp., and *Echium* spp. were found in all the honey samples (100%) with mean values of 14.7 ± 0.5%, 11.3 ± 0.7% and 6.0 ± 0.3%, respectively. The pollen analysis confirmed that all the honeys were monofloral heather honeys, as indicated by the beekeepers. Monofloral status generally refers to the presence of a single pollen type in quantities greater than 45% of the total pollen content in the spectrum. For honey samples having under-represented pollen grains, (*i.e.*, *Thymus*, *Rosmarinus*, *Citrus*, and *Arbutus*), the “monofloral” classification is achieved with a pollen frequency percentage of only 10–20%. However, for honey types characterized by over-represented pollen grains, (*i.e.*, *Castanea*, *Cynoglossum* and *Myosotis*) the “monofloral” classification may be achieved with a pollen frequency percentage of 70–90%. In Europe more than 100 botanical species can give unifloral honeys. Heather honey is produced in Portugal from *Erica* sp., while in Spain and France it comes from either *Calluna* or *Erica* sp. This honey is characterized by its dark brown color, strong flavor and a slightly salty taste. Consumers in Portugal and Central Europe prefer dark honeys, such as heather honeys, and they are more costly than others and in higher demand from the consumer, which means that they also have a higher commercial value for the producers [24].

### 2.2. Phenolics and Flavonoids

Honey is rich in phenolic acids and flavonoids, which exhibit a wide range of biological effects and act as natural antioxidants [34]. The phenolic compounds include flavonoids (chrysin, pinocembrin, pinobanksin, quercetin, kaempferol, luteolin, galangin, apigenin, hesperetin, myricetin), phenolic acids (caffeic, coumaric, ferrulic, ellagic, chlorogenic), stilbenes, lignans, tannins and oxidised polyphenols. This group displays a large diversity of structures; however some of them may not be detected by the common methodologies of analysis carried out by high performance liquid chromatography coupled to distinct detection devices. This is due to the existence of isomers, the difficulty in the separation of some compounds, and the lack of commercial standards. In this context, the preferred methodology is the Folin-Ciocalteu assay, despite the reactions that may occur between the reagent, which is a mixture of phosphotungstic acid and phosphomolibdic acid, and non-phenolic reducing compounds, which may lead to an overestimation of the polyphenol content [35]. 

In the present study the concentration of total phenolic compounds ranged between 718.92 ± 4.41 and 630.89 ± 5.21 mg GAE kg^−1^, the mean value was 688.51 ± 15.57 mg GAE kg^−1^. Concerning the flavonoids, the mean concentration was 525.10 ± 24.44 mg CAE kg^−1^. The minimum value obtained for the latter compounds was 450.72 ± 5.67 and the maximum was 673.98 ± 4.33. The low values of the standard deviation reveal that the precision of this determination was satisfactory.

Total polyphenols found in different honey types ranged from 56–500 mg kg^−1^ [36]. Since different plants contain different phenolic and flavonoid compounds and show fluctuation in their concentration due to climate and soil conditions, the significant variation among the honey samples in their total phenolic and flavonoid contents is due to the variation in their floral sources [17]. Dark honey samples, such as heather honeys, have a significantly higher content of extracted phenolic compounds than clear honey samples [10,35].

### 2.3. Quantification of the Hydrogen Peroxide

The concentration of H_2_O_2_ was determined in the absence and in the presence of catalase. The concentration of this molecule in honey without catalase varied between 2.53 ± 0.97 mM and 4.25 ± 0.88 mM, with a mean value of 3.42 ± 1.11 mM. After the addition of catalase in the honeys, the mean value decreased to 0.18 ± 0.08 mM, suggesting that the inactivation of the H_2_O_2_ was efficient.

Catalase contains four porphyrin heme groups that allow the decomposition of hydrogen peroxide into water and oxygen. Just as in the present survey, studies on the amount of H_2_O_2_ in honey found great differences between the samples, and, consequently large values of standard deviations. This cannot be due to experimental error, but must be due to different factors, among which we should highlight the metalic ions content and the concentration of antioxidants such as the phenolic compound. It has been reported that exposure to excess heat or light also promotes the degradation of H_2_O_2_. Another explanation for the variation of this compound in honeys could be differences in the levels of glucose oxidase [13,37,38]. 

### 2.4. Antimicrobial Activity

The antimicrobial activity of the honey samples was tested against four bacteria and four yeasts species. *B. cereus* was selected because it is an opportunistic pathogen that is a common cause of food poisoning. The other bacterial species were used because, according to the Centers for Disease Control and Prevention (Atlanta, GA, USA), they are the most common pathogens that cause nosocomial infections. These pathogenic microorganisms have developed multidrug resistance because of the indiscriminate use of antimicrobial drugs. This has led to the urgent need for new therapeutic alternatives. Regarding the yeasts used, and despite the great importance of *C. albicans*, the rate of candidaemia caused by non-albicans species is increasing [39]; three species of the *Candida* genus were selected. Another important pathogenic fungus is the encapsulated yeast *C. neoformans*, which is responsible for infectious diseases in patients with AIDS [40].

**Figure 1:**
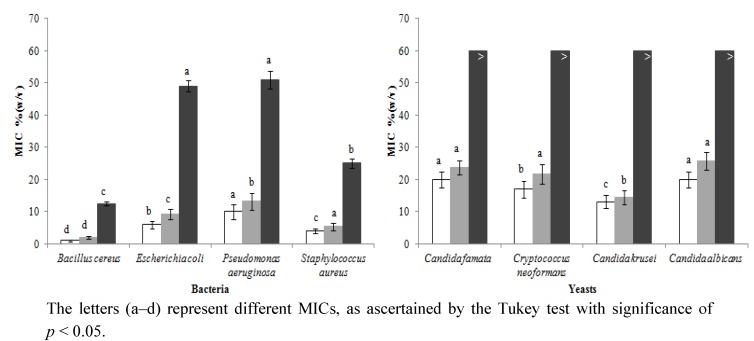
Minimum inhibitory concentration of honey (n = 25): without catalase (□), with catalase (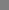), artificial honey (■); against four bacteria (*Bacillus cereus* sp., *Escherichia coli* sp., *Pseudomonas aeruginosa* sp. *and Staphylococcus aureus* sp.) and four yeasts (*Candida famata* sp., *Cryptococcus neoformans* sp., *Candida krusei* sp. *and Candida albicans* sp.).

Figure 1 shows the results for the antimicrobial activity screening of heather honey in the presence and absence of catalase, and the minimum inhibitory concentration (MIC) of artificial honey against Gram positive bacteria (*B. cereus* sp., *S. aureus* sp.), Gram negative bacteria (*E. coli* sp. and *P. aeruginosa* sp.) and yeasts (*C. famata* sp., *C. neoformans* sp., *C. krusei* sp., *C. albicans* sp.). 

The letters (a–d) represent different MICs, as ascertained by the Tukey test with significance of *p* < 0.05.

The MIC was used as a parameter of the significant inhibitory effects induced by honey in the growth of the tested microorganisms. The natural products tested evidenced antimicrobial activity against everything, and showed different selectivity and MICs for each microorganism. The MIC for bacteria, in the absence of catalase, ranged from 1.01 ± 0.50% (w/v) (*B. cereus* sp.) to 10.00 ± 4.72% (w/v) (*P. aeruginosa*). In the presence of catalase, the minimum MIC was 2.00 ± 0.94% (w/v), for *B. cereus* sp. and the maximum was 13.27 ± 5.23% (w/v), for *P. aeruginosa.* Regarding the artificial honey, the inferior MIC value was obtained for *B. cereus sp*. [12.50 ± 1.22% (w/v)] and the highest for *P. aeruginosa* [51.02 ± 5.61% (w/v)]. Using the Tukey test (*p* < 0.001), significant differences among all the bacteria tested were observed. Concerning yeasts, the most sensitive one to the honeys’ action, in the absence and in the presence of catalase, was *C. krusei* sp. [13.16 ± 4.08% (w/v) and 14.95 ± 4.16% (w/v), respectively]. The most resistant one was *C. albicans* sp. [20.00 ± 5.09% (w/v), in the absence of catalase and 25.67 ± 5.50% (w/v), in the presence of that enzyme], followed by *C. famata* sp. The MIC was statically different for all the yeasts, except for *C. famata* and *C. albicans*.

It was found that heather honey has wide antibacterial activity, but limited antifungal activity. For all the assays (involving honey, honey with catalase and artificial honey), the ascending order of resistance was *B. cereus < S. aureus < E. coli* < *P.* *aeruginosa*, for bacteria. Gram positive bacteria evidenced higher susceptibility to the honeys than the Gram negative ones. Indeed, Gram negative bacteria possess an outer membrane and a unique periplasmic space not found in Gram positive bacteria. The resistance of these bacteria towards antibacterial substances is related to the hydrophilic surface of their outer membrane (rich in lipopolysaccharides). It presents a barrier to the penetration of numerous antibiotic molecules and is also associated with the enzymes in periplasme space that break down the molecules introduced from the outside. The ascending order of resistance for yeasts was *C. krusei < C. neoformans < C. famata < C. albicans*, in all the assays except for the ones involving artificial honey, in which efficiency was very similar and reduced.

The information available in the literature about the antimicrobial factors of honey is incomplete and, sometimes, controversial. Table 1, Table 2 summarise the results obtained for the MIC, considering the different microorganisms studied, and the presence of catalase. There was a significant difference among bacteria, and also among yeasts, *P. aeruginosa* had the greatest (11.63 ± 5.23) MIC, and was the most resistant to the honey effect, followed by *E. coli* (7.60 ± 3.69), then *S. aureus* (4.73 ± 2.09), and finally *B. cereus* (1.51 ± 0.90), was the most sensitive to the honey effect. Regarding yeasts, *Candida* presented the highest MIC (23.33 ± 10.31 and 22.38 ± 9.14, for *C. albicans* and *C. famata*, respectively), and they were the most resistant to the honey effect, followed by *C. neoformans* (19.33 ± 8.15), and finally *C. krusei* (14.33 ± 5.68), was the most sensitive to the honey effect. 

**Table 1:** Effect of the bacteria species, presence of catalase, and their interaction on the Minimum Inhibitory Concentration (MIC).

Variable		MIC % (w/v)
Microorganisms	*B. cereus*	1.51 ± 0.90 d
	*S. aureus*	4.73 ± 2.09 c
	*E. coli*	7.60 ± 3.69 b
	*P. aeruginosa*	11.63 ± 5.23 a
Catalase	Absence	5.25 ± 4.29 b
	Presence	7.48 ± 5.47 a
Effects significance	Microorganisms	***
	Catalase	***
	Interaction	NS

The letters (a–d) represent which MICs are different for microorganisms, and for catalase by Tukey test with significance of *p* < 0.001. *** *p* < 0.001. NS—Not significant.

**Table 2:** Effect of the yeasts species, presence of catalase, and their interaction on the Minimum Inhibitory Concentration (MIC).

Variable		MIC % (w/v)
Microorganisms	*C. krusei*	14.33 ± 5.68 c
	*C. neoformans*	19.33 ± 8.15 b
	*C. famata*	22.38 ± 9.14 a
	*C. albicans*	23.33 ± 10.31 a
Catalase	Absence	17.52 ± 7.30 b
	Presence	22.17 ± 10.32 a
Effects Significance	Microorganisms	***
	Catalase	***
	Interaction	NS

The letters (a–c) represent which MIC are different for microorganisms, and for catalase, as ascertained by the Tukey test with significance of *p* < 0.001. *** *p* < 0.001. NS—Not significant.

The effect of the catalase on the MIC was highly significant for all the microorganisms under study. Catalase speeds up the breakdown of hydrogen peroxide into H_2_O and O_2_. It was found that the antimicrobial activity of the catalase-treated honeys was inferior. The effect of the interaction microorganisms x catalase was not statistically significant, suggesting that the differences of the MICs between the microorganisms are identical in the presence or absence of catalase. 

Table 3, Table 4 show the correlations between MIC and polyphenols concentration, and the correlation between MIC and sugars content for all the microorganisms studied. The correlations between MIC x flavonoids, MIC x phenols and MIC x sugars were low and not statistically significant, for the microorganisms studied. 

**Table 3:** Pearson’s correlation coefficients between MIC and phenols, between MIC and flavonoids, and between MIC and sugars for the bacteria studied.

Correlation	*B. cereus*	*S. aureus*	*E. coli*	*P. aeruginosa*
MIC *versus* phenols	−0.115	−0.098	0.043	0.111
MIC *versus* flavonoids	0.365 ***	−0.146	0.089	0.010
MIC *versus* sugars	−0.066	0.177	−0.103	−0.030

*** Correlation is significant for *p* < 0.001.

**Table 4:** Pearson’s correlation coefficients between MIC and phenols, between MIC and flavonoids, and between MIC and sugars for the yeasts studied.

Correlation	*C. krusei*	*C. neoformans*	*C. famata*	*C. albicans*
MIC *versus* phenols	−0.109	−0.117	0.011	−0.080
MIC *versus* flavonoids	0.026	−0.087	0.309 **	0.1356
MIC *versus* sugars	−0.130	−0.113	−0.068	−0.067

** Correlation is significant for *p* < 0.01.

With this work we intended to elucidate the factors underlying the antimicrobial activity induced by the heather honey. Indeed, the applicability of honey as antimicrobial agent requires safe preparations, knowledge of the composition, of the antimicrobial factors and standardized activity [38]. Antimicrobial activity is due both to the physical characteristics (pH, osmolarity) and the properties of H_2_O_2_ and non-peroxide components (lysozyme, organic acids, volatile compounds, phenolic acids, flavonoids, 1,2-dicarbonyl compound methylglyoxal and bee peptides such as apidaecins, abaecin, hymenoaptaecin and defensin) [26,36,37,41,42].

The great variety of information concerning the antimicrobial agents of honey may be attributed to differences in the botanical and geographical origin, and consequently in the chemical composition [21]. The use of limited and non-standardized methods in the determination of the antimicrobial activity can also hamper the comparison of results. However since there are a number of studies indicating appreciable differences among antimicrobial potency of honey the full characterization of antimicrobial potency of honey of diverse origins still appears to be a sound research priority to obtain a reliable data on this valuable beehive product [43]. Many factors are known to affect the physicochemical composition of honey, including climate, geography, apicultural practices and the genetic composition of the plant and bee species.

We can assume that research on honey is still in the phase in which further work will produce increased complexity rather than simplification. For example, for a long time methylglyoxal was thought to be the major bactericidal factor in manuka honey, but recent research has shown us that after neutralization of this compound, manuka honey retained bactericidal activity due to several unknown factors [38].

## 3. Experimental

### 3.1. Chemicals and Apparatus

2,3,5-Triphenyltetrazolium chloride (TTC), dimethyl sulfoxide (DMSO), NaCl, glycerol, catalase from bovine liver (2000–5000 units mg^−1^ protein) and (+)-Catechine (CA) were obtained from Sigma Chemical Co. (St. Louis, MO, USA). 3,4,5-Trihydroxybenzoic acid (gallic acid; GA), aluminium chloride (AlCl_3_), NaNO_2_ and NaOH were purchased from Acros Organic (Geel, Belgium). The Folin–Ciocalteu reagent (FCR), gentamicin and sodium carbonate (Na_2_CO_3_) were obtained from Merck (Darmstadt, Germany). Methanol (MeOH) was obtained from Pronolab (Lisboa, Portugal). Mueller Hinton (MH) and Nutrient Broth (NB) were purchased from Himedia laboratories Pvt. Ltd., (Mumbai, India). High purity water (18 MΩ cm), which was used in all experiments, was obtained from a Milli-Q purification system (Millipore, Bedford, MA, USA). Spectrophotometric measurements were made using a Unicam Helios AlphaUV–visible spectrometer (Thermo Spectronic, Cambridge, UK). For the determination of the H_2_O_2_ concentration, an *o*-dianisidine (3,3' dimethoxybenzidine) assay was used (Sigma-Aldrich, St. Louis, MO, USA). A Leitz Diaplan microscope (Leitz Messtechnik GmbH, Wetzlar, Germany) was used for pollen identification.

### 3.2. Honey Samples

Twenty-five (n = 25) honey samples, from *Apis mellifera*, were collected by beekeepers from different apiaries, located in Portugal, according to European apiculture standards. A single 250 g jar of honey was delivered to the Microbiology Lab, where it was stored in a dark place at room temperature (±20 °C) until analysis, which occurred no more than two months after the extraction from the hives by beekeepers. All the honey samples showed no sign of fermentation or spoilage.

### 3.3. Sample Floral-Type Identification

Even though the beekeepers themselves, according to the best of their knowledge and the location of hives, declared the product as monofloral heather honeys, all the samples were subjected to qualitative pollen analysis as per Erdtman’s acetolysis method, reported previously in detail [44,45]. The aim of that analysis was to confirm that analysed samples could be declared as heather monofloral honey. The examination of the pollen slides was carried out at ×400 and ×1000. A minimum of 1000 pollen grains was counted per sample. In order to recognise the pollen grains, we used the reference collection of the CIMO-Mountain Research Center (Agricultural College of Bragança) and different pollen morphology guides. The following terms were used for pollen frequency classes: predominant pollen (P, more than 45% of pollen grains counted), secondary pollen (S, 16–45%), minor important pollen (M, 3–15%) and isolated pollen (I, 1–3%).

### 3.4. Bioactive Compounds Quantification

#### 3.4.1. Total Phenolic Content (TPC)

The total phenolic content in the honey was recorded by the Folin–Ciocalteu method described by [28]. Briefly, a dilute solution of each honey sample in MeOH (500 μL of 1:10 g mL^−1^) was mixed with 500 μL of the FCR and 500 μL of Na_2_CO_3_ (10% w/v). After incubation in the dark at room temperature for 1 h, the absorbance of the reaction mixture at 700 nm is determined against the blank (the same mixture without the honey). GA standard solutions (0.01–0.08 mM) were used for constructing the calibration curve (y = 2.3727x + 0.0022; R^2^ = 0.9998). Total phenols contents were expressed as mg of GA equivalents per kg of honey (GAEs).

#### 3.4.2. Total Flavonoid Content (TFC)

For flavonoid contents the aluminium chloride method was used, as recommended by [35]. Briefly, honey (250 μL) was mixed with distilled H_2_O (1.25 mL) and a 5% NaNO_2_ solution (75 μL). After 5 min, a 10% AlCl_3_·H_2_O solution (150 μL) was added. After 6 min, 1M NaOH (500 μL) and distilled H_2_O (275 μL) were added to the mixture which was vortexed. The intensity of the pink colour of the reaction mixture at 510 nm is determined against the blank (the same mixture without the honey). Catechin (CA) standard solutions (0.022–0.34 mM) were used for constructing the calibration curve (y = 0.9652x − 0.0091; R^2^ = 0.9981). Total flavonoids content (TFC) was expressed as mg of CA equivalents per kg of honey (CAEs).

### 3.5. Hydrogen Peroxide Assay

The concentration of H_2_O_2_ in honey samples was determined before and after the treatment of the samples with catalase, prior to the assessment of the antimicrobial activity. The determination of the hydrogen peroxide was repeated after the addition of catalase to ensure that there were no remnants of this molecule. This assay was carried out according to the procedure described in detail by [38]. In brief, samples of honey (40 μL) were added to reagent (135 μL), consisting of 50 μg mL^−1^ *o*–dianisidine and 20 μg mL^−1^ horseradish peroxidase type IV in 10 mM phosphate buffer pH 6.5. After 5 min. of incubation at room temperature, reactions were stopped by addition of 120 μL 6M H_2_SO_4_and absorption at 540 nm was measured. The color of the reaction was measured by absorbance at 560 nm. H_2_O_2_ concentrations were calculated using a calibration curve of 2-fold serial dilutions of H_2_O_2_ ranging from 2,200 to 2.1 mM. Sterile deionized water and a solution of catalase were used as negative controls. 

### 3.6. Antimicrobial Activity Tests

#### 3.6.1. Microbial Species

In the present study four bacterial and four fungal species, isolated from biological fluids, were used: *Bacillus cereus* ESA (from feces), *Staphylococcus* *aureus* (from chronic wounds), *Escherichia* *coli* ESA 34 (from blood), *Pseudomonas aeruginosa* ESA 567 (from wounds), *Candida krusei* ESA 421(from blood), *Cryptococcus neoformans* ESA 578 (from expectoration),* Candida famata* ESA 99 (from wounds), *Candida albicans* ESA 453(from urine), isolated in the Hospital Centre of the Northeast, Bragança (Portugal), and identified at the Microbiology Laboratory of the Escola Superior Agrária (ESA), Bragança. 

In the present study four bacterial and four fungal species, isolated from biological fluids, were used: *Bacillus cereus* ESA (from feces), *Staphylococcus* *aureus* (from chronic wounds), *Escherichia* *coli* ESA 34 (from blood), *Pseudomonas aeruginosa* ESA 567 (from wounds), *Candida krusei* ESA 421 (from blood), *Cryptococcus neoformans* ESA 578 (from expectoration),* Candida famata* ESA 99 (from wounds), *Candida albicans* ESA 453 (from urine), isolated in the Hospital Centre of the Northeast, Bragança (Portugal), and identified at the Microbiology Laboratory of the Escola Superior Agrária (ESA), Bragança. 

#### 3.6.2. Culture Media and Inoculums

Strains were stored in Mueller–Hinton medium plus 20% glycerol at −70 °C. Before experimental use, cultures from solid medium were subcultivated in liquid media, incubated and used as the source of inoculums for each experiment. The inoculum for the assays were prepared by diluting cell mass in 0.85% NaCl solution, adjusted to 0.5 MacFarland scale, confirmed by spectrophotometrical reading at 580 nm. Cell suspensions were finally diluted to 10^4^ CFU mL^−1^ in order to use them in the biological activity assays.

#### 3.6.3. Antimicrobial Assay

Before this assay, the chemical contaminants present in honey were determined, to eliminate their effect. Antimicrobial tests were carried out according to [46], using Nutrient Broth (NB) or Yeast Peptone Dextrose (YPD) on microplate (96 wells). Each honey sample was divided into two subsamples: (i) honey; (ii) honey to which catalase was added. All the subsamples were diluted in sterile water (60%; w/v) and transferred into the first well, and serial dilutions were performed. The second subsample was treated with catalase from bovine liver (5,000 units mg^−1^) at ratio of 2,700 units mg/solid per 1 g of honey, to destroy any peroxide present so that residual non-peroxide antibacterial activity could be determined. The inoculum was added to all wells and the plates were incubated at 37 °C for 24 h (bacteria) for 48 h (yeast). Gentamicin and fluconazole were used as controls, for bacteria and yeasts, respectively. In each experiment a positive control (inoculated medium) and a negative control (medium) was introduced. Antimicrobial activity was detected by adding 20 µL of 0.5% TTC solution. The Minimum Inhibitory Concentration (MIC) was defined as the lowest concentration of honey that inhibited visible growth of microorganisms used. 

#### 3.6.4. Artificial Honey

An artificial honey solution with a carbohydrate composition similar to natural honey was used to determine whether inhibitory effects were due to the sugar content of the honey samples. In the preparation of 100 g of synthetic honey, 1.5 g sucrose, 7.5 g maltose, 40.5 g d-fructose, and 33.5 g d-glucose were dissolved in 17 mL of sterile, deionized water. This product was submitted to antimicrobial assays, using the procedure described in the Section 3.6.3*.*

### 3.7. Statistical Analysis

Each honey sample was analyzed in triplicate. Prior to the statistical analysis, normality tests (Shapiro-Wilk, Anderson-Darling, Liliefors and Jarque-Bera) were carried out. In each parameter, the differences between honeys were analyzed using one-way analysis of variance (ANOVA) followed by Tukey’s HSD Test with α = 0.05. This treatment was carried out using SAS v. 9.1.3 program. Results are shown as mean values and standard deviation. A Pearson’s correlation test was performed to analyze if phenols and flavonoids, H_2_O_2_ and sugars were correlated with MIC for each microorganism.

## 4. Conclusions

In the present study a detailed analysis of the antimicrobial activity of heather honey was carried out. All the microorganisms under study were inhibited by honey (with or without catalase), the most important factor from a total of four analyzed was H_2_O_2_. The yeasts were more resistant than bacteria. Raw honey or isolated honey-derived components might be of great value for treatment of infections caused by antibiotic-resistant bacteria. The use of novel and powerful high-throughput techniques that are currently used in drug development will be of value to ascertain the medical properties of honey. Once the compound’s structure is known, the chemical can serve as a prototype or “lead compound” for designing more effective therapeutic agents of similar chemical structure. Therefore, detailed information should be obtained concerning the functionality of honeys of different origin to enable their use in the health services. More studies will be essential to further elucidate how the action mechanisms are carried out in the cells.
